# Sea–Sky Line and Its Nearby Ships Detection Based on the Motion Attitude of Visible Light Sensors

**DOI:** 10.3390/s19184004

**Published:** 2019-09-16

**Authors:** Xiongfei Shan, Depeng Zhao, Mingyang Pan, Deqiang Wang, Lining Zhao

**Affiliations:** 1 Navigation College, Dalian Maritime University, Dalian 116026, China; shanxiongfei@dlmu.edu.cn (X.S.); dpzhao@dlmu.edu.cn (D.Z.); dqwang@dlmu.edu.cn (D.W.); zhaolining@dlmu.edu.cn (L.Z.); 2 Vessel Navigation System National Engineering Research Center, Dalian Maritime University, Dalian 116026, China

**Keywords:** SSL, six-degrees-of-freedom motion, motion attitude model, edge detection, straight-line fitting, visual saliency

## Abstract

In the maritime scene, visible light sensors installed on ships have difficulty accurately detecting the sea–sky line (SSL) and its nearby ships due to complex environments and six-degrees-of-freedom movement. Aimed at this problem, this paper combines the camera and inertial sensor data, and proposes a novel maritime target detection algorithm based on camera motion attitude. The algorithm mainly includes three steps, namely, SSL estimation, SSL detection, and target saliency detection. Firstly, we constructed the camera motion attitude model by analyzing the camera’s six-degrees-of-freedom motion at sea, estimated the candidate region (CR) of the SSL, then applied the improved edge detection algorithm and the straight-line fitting algorithm to extract the optimal SSL in the CR. Finally, in the region of ship detection (ROSD), an improved visual saliency detection algorithm was applied to extract the target ships. In the experiment, we constructed SSL and its nearby ship detection dataset that matches the camera’s motion attitude data by real ship shooting, and verified the effectiveness of each model in the algorithm through comparative experiments. Experimental results show that compared with the other maritime target detection algorithm, the proposed algorithm achieves a higher detection accuracy in the detection of the SSL and its nearby ships, and provides reliable technical support for the visual development of unmanned ships.

## 1. Introduction

In recent years, with the continuous development of artificial intelligence (AI), big data, and communication technology, unmanned driving technology has made breakthrough achievements. Unmanned aerial vehicles (UAVs) have gradually entered the civil field from the military field, and unmanned ground vehicles (UGVs) are continually testing on public roads around the world. The research on unmanned ships is also developing rapidly. Major research institutions at home and abroad are investing a large amount of manpower, material resources, and financial resources to carry out theoretical research, technology research, and development of large-tonnage unmanned merchant ships. The key technologies of unmanned ships mainly include situational awareness, intelligent decision-making, motion control, maritime communication, and shore-based remote control, etc., and situational awareness is the premise of all other technologies. The advanced sensors are used to obtain the situation information around unmanned ships, provide basic data support for complex tasks such as intelligent decision-making and motion control, and ensure the autonomous operation safety of unmanned ships [1].

Currently, ships perceive the maritime environment mainly through two kinds of sensors, namely, radio detection and ranging (RADAR) and automatic identification system (AIS). They transmit the target information to the electronic chart display and information system (ECDIS), which realizes a certain degree of intelligent analysis and decision. However, the maritime navigation environment is complex and variable. RADAR and AIS cannot directly reflect the spatial information of detection targets. The situational awareness cannot be established quickly, and mariners need to confirm the situation. At the same time, RADAR detection is sensitive to meteorological conditions and the shape, size, and material of the target. AIS cannot effectively detect small targets that are not equipped with it or are not turned on. Visible light sensors are intuitive, reliable, informative, and cost-effective [2]. With the continuous development of computer vision technology, visible light cameras as important situational awareness sensors are gradually being applied to unmanned ships, providing a reliable source of information for intelligent decision-making.

The main targets for maritime detection using cameras include ships, rigs, navigation aids, and icebergs. When maritime targets appear in the field of view of the camera, they must appear in the vicinity of the sea–sky line (SSL). As the distance between the camera and the target approaches, the target gradually enters the sea area. It can be seen that extracting the SSL and performing maritime target detection in its vicinity can greatly reduce the target detection range and reduce the complexity and calculation amount of the algorithm. However, a target near the SSL has a very small area in the image, usually only a few tens or hundreds of pixels, which is easily overwhelmed by the complex sea–sky background, resulting in target missed detection or false detection [3]. Therefore, this paper proposes an algorithm based on the motion attitude model of a visible light camera for the SSL and its nearby ships.

## 2. Related Work

In general, maritime target detection technology mainly includes three steps, namely, SSL detection, background removal, and foreground segmentation. Based on the research status at home and abroad in recent years, this paper briefly reviews the three algorithms and proposes the main technical framework.

### 2.1. SSL Detection

The SSL is an important feature of maritime images, and there are many related researches, which are mainly divided into two categories. The first category is based on the combination of edge detection and straight-line fitting. The image is processed by the edge detection operator, and then the high gradient edge pixels are straight-line fitted or projected. Liu [4] proposed an SSL detection algorithm based on inertial measurement and Hough transform fusion. The inertial data of the shipboard camera are used to estimate the position of the SSL in successive frames, then Canny operator and Hough transform are used to realize SSL extraction in the detection region. Wang Bo et al. [5] proposed an SSL detection algorithm based on gradient saliency and region growth. The gradient saliency calculation effectively improves the characteristics of the SSL and suppresses the influence of complex sea conditions such as clouds and sea clutter. Kim et al. [6] proposed an algorithm for estimating the position of the SSL by camera pose and fitting it using random sampling consistency (RANSAC). Fefilatyev et al. [7] used the combination of Gaussian distribution and Hough transform to select the optimal SSL from five candidate SSLs. Santhalia et al. [8] proposed a Sobel operator edge detection algorithm based on eight directions, which effectively eliminates edge noise and has small computational complexity and strong stability. The second category is based on the method of image segmentation, which extracts the upper part of the SSL by threshold processing or background modeling. Dai et al. [9] proposed an edge detection algorithm based on local Otsu segmentation, which solves the problem of poor global threshold segmentation. Zhang et al. [10] proposed an SSL extraction algorithm based on Discrete Cosine Transform (DCT) coefficients. The image is segmented into 8 × 8 non-overlapping blocks, and the DCT coefficients in the block are calculated to segment the sky and sea areas. Zeng et al. [11] extracted the contour edges using the improved Canny operator of the surrounding texture suppression, and then voted the Hough transform to finely detect the horizontal or oblique SSL. Nasim et al. [12] proposed a K-means algorithm to segment the sea scene into clusters, and extract the SSL by analyzing the image segments. The above algorithms have achieved good detection results in their respective experiments, but the first category of algorithm is not able to balance SSL edge extraction and wave edge suppression according to the gradient extraction edge process, and the second category of algorithm is not able to get the SSL limited by the image segmentation accuracy. 

### 2.2. Background Removal

In the maritime scene, we usually segment the sea–sky background by simulating the color, texture, saturation, and other features, and subtract it from the original image. Kim et al. [13] used improved mean difference filtering to improve the target signal-to-noise ratio while processing infrared images, and averaged the sea–sky background to remove sea clutter interference. However, this method only worked well for structural clutter similar to the SSL, and had a poor effect on the sea surface interference with strong light reflection. Zeng et al. [14] used the surrounding texture filter instead of mean-shift filter to improve the mean-shift image segmentation algorithm, and controlled the filter parameters to perform fast region clustering to remove the sea–sky background. However, the texture filter parameters and clustering parameters needed to be manually set, which needed a certain prior knowledge. In addition, a technique based on the visual saliency model is gradually being applied to maritime target detection. It simulates human visual features through intelligent algorithms, suppresses the sea–sky background, and extracts visual salient regions in the image—that is, regions of human interest. Fang et al. [15] applied the theory of color space and wavelet transform to extract the low frequency, high frequency, hue, saturation, and brightness characteristics of the task water image. The visual attention operator was used to fuse various features, effectively overcoming the background disturbances such as waves, wakes, and onshore buildings. Lou et al. [16] solved the small target detection problem in color images from two aspects of stability and saliency. By multiplying the stability and saliency maps by pixels, the noise interference in the background was eliminated. Agrafiotis et al. [17] designed a maritime tracking system by combining a visual saliency model with a Gaussian Mixed model (GMM) and used an adaptive online neural network tracker to further refine the tracking results. Liu et al. [18] achieved further enhancement of the visual saliency model through a two-scale detection scheme. On a larger scale, the sea surface was removed by the mean-shift filter. On a smaller scale, the target was coarsely extracted by extracting the edge of the significant region, and then the fine processing of the chroma component was used to select the output target. The above algorithm achieves background removal by reducing the background noise of the sea–sky background and enhancing the salient features of the region of interest, but when there is strong cloud, wave, or ship wake disturbance in the sea–sky background, the saliency is the same or higher than the target, which causes the visual saliency algorithm to produce large errors. In addition to the above documents, Ebadi et al. [19] proposed a modified approximated robust PCA algorithm that can handle moving cameras and takes advantage of the block sparse structure of the pixels corresponding to the moving objects.

### 2.3. Foreground Segmentation

After the image background is removed, we can apply morphological processing to obtain the maritime target. Westall et al. [20] applied improved morphological processing of close-minus-open (CMO) techniques to enhance target detection. Fefilatyev [21] used the Otsu algorithm to obtain global thresholds in the region above the SSL, and used the global threshold to segment the target vessel. Although features such as edges and contours are widely used in target ship detection, it is still difficult to achieve ideal results in complex sea–sky backgrounds with the above algorithms. Besides, Kumar et al. [22] and Selvi et al. [23] made full use of the target ship’s color, texture, shape, and other information, and used the support vector machine to classify the target. Frost et al. [24] also applied the prior knowledge of ship shape to the level set segmentation algorithm to improve ship detection results. Loomans et al. [25] integrated a multi-scale Histogram of Oriented Gradient (HOG) detector and a hierarchical Kanade-Lucas-Tomasi (KLT) feature point tracker to track ships in the port, and achieved better detection and tracking effects. The above algorithm is not based on the background subtraction algorithm, but is based on the manually set ship characteristics for target detection. With the continuous development of deep learning technology, the feature extraction algorithm, based on convolutional neural network, is gradually dominating image classification, detection, segmentation, etc., and is gradually being applied to the field of maritime target detection. Ren et al. [26] proposed an improved Faster R-CNN system to detect small target ships in remote sensing images. The statistical algorithms were used to screen the appropriate anchors, and the detection techniques were greatly improved by using jump links and texture information. Yang et al. [27] designed a rotational density pyramid network model to extract the ship’s direction while accurately detecting the target ship. Zhang et al. [28] proposed a scheme combining saliency detection and convolutional neural networks to accurately detect ships in remote sensing images of different poses, scales, and shapes. In addition to the above documents, Biondi [29] presented a complete procedure for the automatic estimation of maritime target motion parameters by evaluating the generated Kelvin waves detected in synthetic aperture radar (SAR) images. Graziano et al. [30] proposed a novel technique using X-band Synthetic Aperture Radar images provided by COSMO/SkyMed and TerraSAR-X for ship wake detection. Biondi et al. [31] proposed a new approach where the micro-motion estimation of ships, occupying thousands of pixels, was measured, processing the information given by sub-pixel tracking generated during the co-registration process of several re-synthesized time-domain and overlapped sub-apertures. 

**Figure 1:**
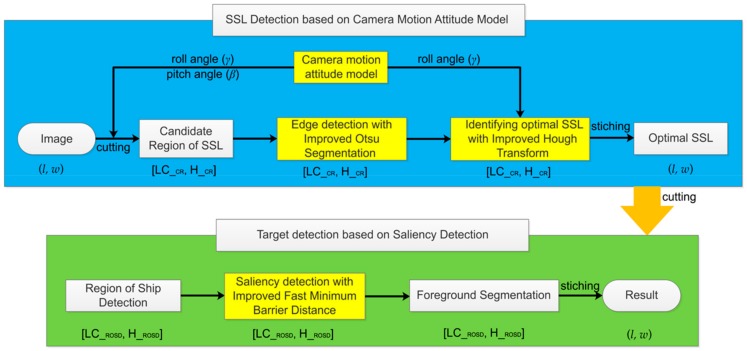
Technical framework of this paper. Roll angle (γ) and pitch angle (β) represent inertial data, (*l, w*) represents the size of the original image. [LC_CR, H_CR] represents the candidate (CR) of the image, where LC_CR represents the upper left corner of the CR, and H_CR represents the height of the CR. [LC_ROSD, H_ROSD] represents the region of ship detection (ROSD) of the image, where LC_ROSD represents the upper left corner of the ROSD, and H_ROSD represents the height of the ROSD.

In summary, the above algorithms have achieved good application results in their respective research fields, but it is still difficult to achieve high detection accuracy for the SSL and its nearby ships in the complex sea–sky background. For the above problems, we propose the technical framework of this paper, which mainly includes two technologies, as shown in Figure 1. First, SSL detection. After the camera and the inertial sensor acquire the data synchronously, we pass the inertial data to the camera motion attitude model to obtain the image candidate region (CR) position, then cut the CR from the original image, and only perform edge detection and Hough transform in the CR to extract the optimal SSL. Finally, the CR with the optimal SSL is stitched back to the original image. Second, according to the optimal SSL position, we cut the region of ship detection (ROSD) of the image, and then only perform saliency detection and foreground segmentation on the ROSD, and finally stitch the detection result back to the original image.

In the remainder of this paper, we describe the camera motion attitude model in Section 3, the SSL detection model in Section 4, and the visual saliency detection model in Section 5. We introduce the dataset used in the experiment and compare experiments with other algorithms in Section 6. Finally, in Section 7, we summarize and draw conclusions. 

## 3. Camera Six-Degrees-of-Freedom Motion Attitude Modeling

**Figure 2:**
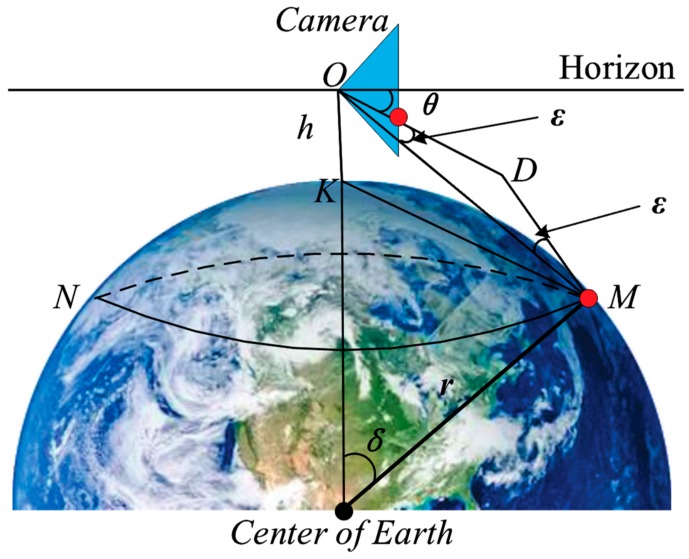
Geometric relationship between the big circle and the camera position.

In navigation, the ship is sailing in a large circle at sea; the tester with an eye height of *h* sees that the farthest sea and the sky intersect into a circle, which is called the tester’s visible horizon, that is, the SSL. In ship vision, we use cameras instead of human eyes for sea target detection and identification. Assuming that the installation position of the camera is *h* from the sea level, the geometric relationship can be obtained considering the curvature of the earth and the difference of atmosphere refraction, as shown in Figure 2. The circle *MN* represents the SSL and the blue triangle represents the camera. Before using it, we finished camera calibration and distortion correction [32]. Therefore, in this analysis, we suppose the optical axis of the camera is parallel to the horizontal plane, which is called the initial state of the camera motion. The point *O* is the camera center, the point *K* is the projection of the point *O* at the sea level, *r* represents the radius of the earth, δ represents the angle of the ball, ε represents the difference of atmosphere refraction, the difference in the navigation is (1/13) δ, and the straight line *OM* represents the actual distance from camera to the SSL instead of $\hat{KM}$, which is expressed by $D_{e}$. In the triangle ∆OKM, since both $\left(\delta /2\right)$ and $\left(\delta /2-\varepsilon \right)$ are small angles, we can approximate $\cos\left(\delta /2\right)\approx 1$ and $\sin\left(\delta /2-\varepsilon \right)\approx \delta /2-\varepsilon \;$, and $D_{e}$ can be obtained by Equation (1). According to the 1 nautical mile representing 1852 meters in navigation, it can be inferred that the *r* is 6366707 m. The position angle θ of the SSL in the camera can be obtained by Equation (2).

(1) $$D_{e}=\frac{h\cos\left(\delta /2\right)}{\sin\left(\delta /2-\varepsilon \right)}\approx \sqrt[{}]{\frac{26rh}{11}}=3871\sqrt[{}]{h}$$

(2) $$\theta =\pi /2-\varepsilon -∠KOM=\frac{11D_{e}}{13r}=0.0295\sqrt{h}$$

In order to simplify the projection relationship of the camera, we assume the sea level as a plane, while ignoring the relative motion of the camera and the ship, so that the camera coordinate system coincides with the ship’s motion coordinate system. Next, we model the camera’s six-degrees-of-freedom motion and the SSL position according to the coordinate system projection method [33].

### 3.1. Influence of Camera Swaying, Surging, and Yawing Motions on the Position of the SSL

Under the condition of maintaining the initial state, the height *h* of the camera remains unchanged when the camera only performs the swaying, surging, and yawing motions. It can be known from Equation (1) that θ is only related to *h*, so the camera swaying, surging, and yawing motions have no effect on the position of the SSL on the imaging plane.

### 3.2. Influence of Camera Heaving and Pitching Motions on the Position of the SSL

**Figure 3:**
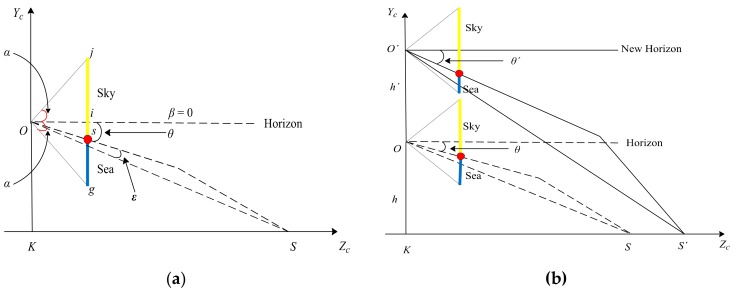
Geometric relationship between the sea–sky line (SSL) position and the camera position. (**a**) Geometric relationship under the initial state. (**b**) Geometric relationship under the camera heaving motion.

Under the condition of maintaining the initial state, we assume the sea level as a plane according to Figure 2, and obtain the geometric relationship as shown in Figure 3a. The triangle represents the camera. In the imaging plane of the camera, the line *js* represents the sky area, the line *sg* represents the sea area, the point *s* represents the projection point of the SSL, and the point *i* represents the center point, which is also taken as the origin (0, 0) of the image coordinate system. Assuming that the pitch angle of the camera is represented by β, the camera’s vertical viewing angle is represented by 2 α, the longitudinal width of the imaging plane of the camera is *w*, and the position of the SSL in the image is represented by $z_{s}$, and $z_{s}$ can be obtained by:

(3) $$z_{s}=w\;\left(-\theta \right)/2\alpha$$

#### 3.2.1. Influence of Camera Heaving Motion

Under the condition of maintaining the initial state, when the camera only performs the heaving motion, as shown in Figure 3b, it is assumed that the heaving height is $h^{\prime }$, and the point $O^{\prime }$ represents the new position of the camera center. According to Equation (1), the position angle $\theta^{\prime }$ and the position $z_{h^{\prime }}$ can be obtained by:

(4) $$\theta^{\prime }=0.0295\sqrt{h+h^{\prime }}$$

(5) $$z_{h^{\prime }}=w\;\left(-\theta^{\prime }\right)/2\alpha$$

#### 3.2.2. Influence of Camera Pitching Motion

**Figure 4:**
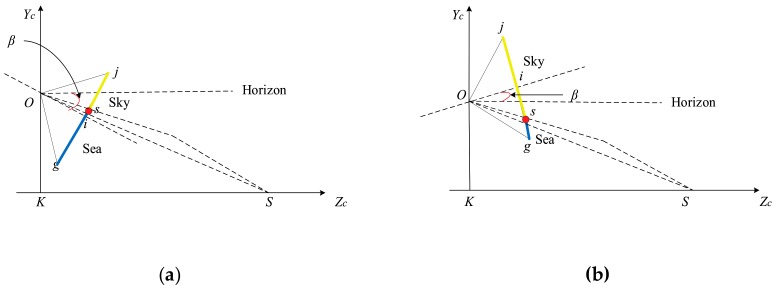
Geometric relationship between the SSL position and camera pitching motion. (**a**) Clockwise rotation. (**b**) Counterclockwise rotation.

Under the condition of maintaining the initial state, when the camera only performs the pitching motion, as shown in Figure 4, it is assumed that the pitch angle β clockwise rotation is positive and the counterclockwise rotation is negative. Under β clockwise rotation, when 0<β<θ, the SSL is located at the lower part of the imaging plane center line and gradually approaches it as β increases. When β=θ, the SSL is located at the center line of the imaging plane. When θ<β<θ+α, the SSL is located on the center line of the imaging plane. As the β increases, it gradually moves away from the center line and close to the top of the image. When β>θ+α, the SSL is not in the imaging plane, and only the sea area can be seen in the image. Under β counterclockwise rotation, when θ−α<β<0, the SSL is located at the lower part of the center line of the imaging plane, and as the β increases, it gradually moves away from the center line and close to the bottom of the image. When β<θ−α, the SSL is not in the imaging plane, and only the sky area can be seen in the image. According to the above analysis, the position of the SSL after the pitching motion can be obtained by:

(6) $$z_{\beta }=\left\{\begin{array}{cc} w\;\left(\beta -\theta \right)/2\alpha & if\;\theta -\alpha <\beta <\theta +\alpha \\ \mathrm{invalid} & \mathrm{else} \end{array}\right.$$

### 3.3. Influence of Camera Rolling Motion on the Position of the SSL

**Figure 5:**
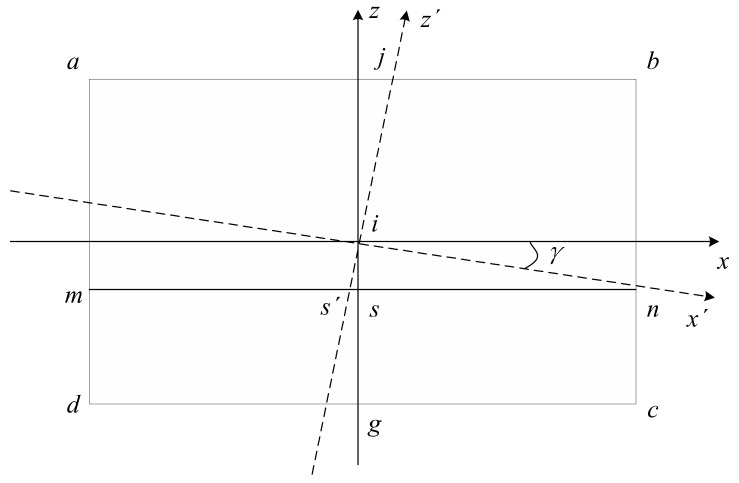
Geometric relationship between the SSL position and camera rolling motion.

Under the condition of maintaining the initial state, when the camera only performs the rolling motion, as shown in Figure 5, it is assumed that the rolling angle γ is clockwise rotated (it is the same as γ counterclockwise rotation), $x^{\prime }z^{\prime }$ is a new image coordinate system, and the SSL intersects the $z^{\prime }$ axis at $s^{\prime }$. So, the SSL can be expressed by:

(7) $$y=x\;\tan\gamma +z_{s}/\cos\gamma$$

Comprehensive analysis of the relationship between the camera six-degrees-of-freedom motion and the SSL shows that when the camera performs the swaying, surging, and yawing motions, the SSL does not change in the image coordinate system. However, when the camera performs the heaving and pitching motions, the SSL performs a translational motion up and down in the image coordinate system, and when the ship performs the rolling motion, the SSL performs a rotational motion in the image coordinate system. Equations (1)–(7) can be used to obtain the estimation equation of the SSL in the image coordinate system, as shown in Equation (8), where the range of β is θ−α<β<θ+α. It can be seen from Equation (8) that the height change $h^{\prime }$ generated by the camera’s heaving motion has less influence on the position of the SSL in the image, and it is also much smaller than the installation height of the camera; so, Equation (8) can be simplified to obtain the final SSL estimation equation, as shown in Equation (9).

(8) $$y=x\;\tan\gamma +w/2\alpha \;\left(-0.0295\sqrt{h+\Delta h}\;(1/\cos\gamma )+\left(\beta -0.0295\sqrt{h}\right)\right)$$

(9) $$y=x\;\tan\gamma +w/2\alpha \left(\beta -0.0295\sqrt{h}\left(1/\cos\gamma +1\right)\right)$$

## 4. Edge Detection and Hough Transform Algorithm for the Detection of the SSL

### 4.1. Estimating the CR of the SSL

**Figure 6:**
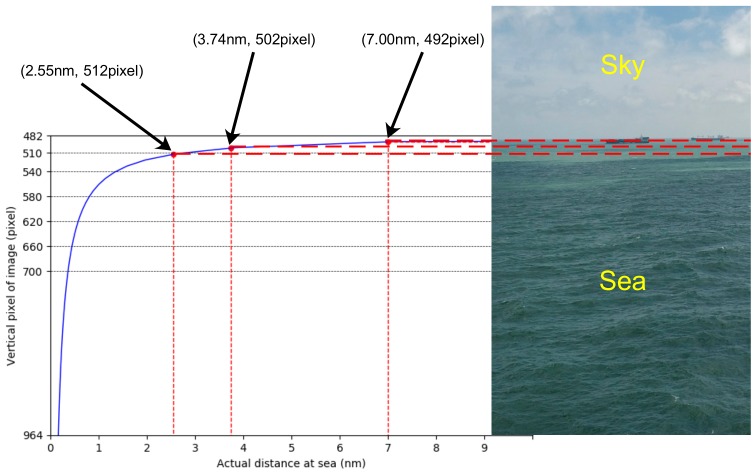
Relationship between the pixel points on the image and the actual distance at sea.

In order to estimate the CR of the SSL, we need to change from the camera coordinate system to the image coordinate system; that is, the coordinate origin moves from the center point to the upper left corner. Then, we begin to explore the relationship between the pixel points on the image and the actual distance at sea. First, we find the position of the SSL on the image in the current coordinate system, as shown in Equation (10), where $z_{i}$ represents the pixel points on the image. Then, through Equations (1) and (10), we can obtain the relationship between $z_{i}$ and the actual distance at sea, as shown in Equation (11). Assuming *h* = 20 m, the camera parameters are *w* = 964 pixels and α = 3.7°, and the relationship between *D* and $z_{i}$ can be obtained as shown in Figure 6, where horizontal and vertical coordinates represent *D* and $z_{i},$ respectively. Since we only want to show the relationship between the SSL and the sea area, the value of the ordinate is from the center of the image to the bottom, so the range is [482, 964]. From Figure 6, it can be seen that the closer to the SSL, the larger the actual distance represented by each pixel. A distance of 2.55 nm or beyond from the camera can be represented by 30 pixels on the image.

**Figure 7:**
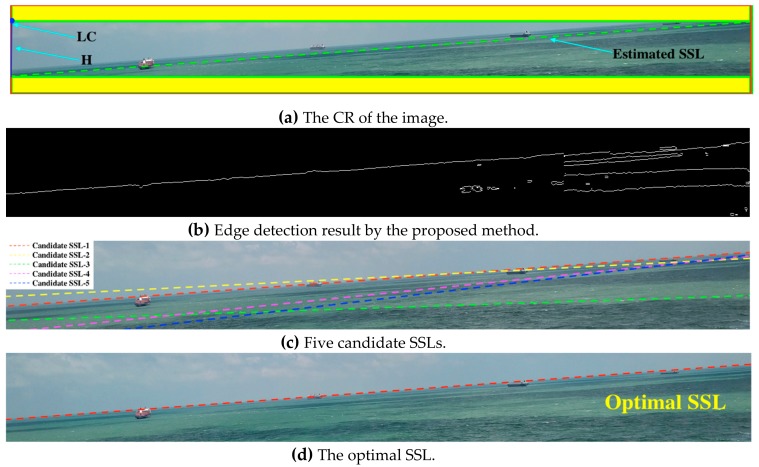
Figures of each stage in the SSL extraction algorithm. In (**a**), the camera parameters are h=20 m, α=3.7°, l=1288 pixels, w=964 pixels; the inertial sensor parameters are $\beta =0^{{}^{\circ}},\;\gamma =5.0^{{}^{\circ}}$; and the green dotted line represents the estimated SSL obtained by the camera motion attitude model. In (**b**), N=16. In (**c**), the clustering parameter is 5. In (**d**), ω = 0.4.

Considering that the SSL is usually a straight line that runs through the entire image and generally has a certain angle of inclination, we use a rectangle to describe the SSL. The upper left corner and the height of the rectangle are represented by *LC* and *H*, respectively. In order to reduce the estimation error, we add a yellow area with a height of 30 pixels to the upper and lower sides of the rectangular area as the CR of the SSL. The parameter value can be obtained by Equation (12), as shown in Figure 7a.

(10) $$z_{i}=w\;\left(\alpha +\theta \right)/2\alpha$$

(11) $$D=\frac{h\cos2.36\left(2\alpha z_{i}/w-\alpha \right)}{\sin(2\alpha z_{i}/w-\alpha )}$$

(12) $$CR=[LC-\left(0,\;30\right),H+60]$$

### 4.2. Edge Detection in the CR

After acquiring the CR, it is only necessary to process the image edges in the region, which can effectively reduce the calculation amount of the image processing work. In this paper, a novel edge detection algorithm based on the local Otsu segmentation is designed in the CR. The specific algorithm is shown as follows:

1. Preprocessing. The CR is grayscaled and smoothed with median filtering to filter out noise. Median filtering is often used to remove salt and pepper noise, which has a good smoothing effect on the sea surface reflected by strong light, and can maximize edge information.
2. Obtaining a binary map by the local Otsu algorithm. According to the gray information of the CR, the Otsu algorithm automatically selects the threshold that maximizes the variance between the two types of pixel as the optimal threshold. For the whole sea–sky image, besides the sea and the sky, there are many other types of pixels, such as clouds, waves, and strong light reflections. It is difficult to obtain an accurate global threshold for the whole image by Otsu algorithm, and the image segmentation accuracy is poor. In this paper, the CR after median filtering is processed into *N* adjacent image blocks. The pixel distribution of the sky and the sea region in most image blocks has significant differences. Then, the Otsu algorithm is applied to each image block to obtain a local binary image, and finally the binarized image blocks are spliced back to the CR.
3. Edge extraction. For the binary image of the CR, we check the position of the pixel mutation in the vertical direction line-by-line, and the position of the pixel mutation is the edge of the binary image, as shown in Equation (13), where $I_{edge}\;$ (·) represents the edge image, $I_{bm}$ (·) represents the binary image, $w_{i}$ represents the position along any line of pixels, and ⨂ represents the morphological XOR operation. The edge extraction effect is shown in Figure 7b.
  (13) $$I_{edge}\left(w_{i},l\right)=I_{bm}\left(w_{i},l\right)⨂\;I_{bm}\left(w_{i+1},l\right)$$

### 4.3. Identifying the Optimal SSL with Improved Hough Transform

**Figure 8:**
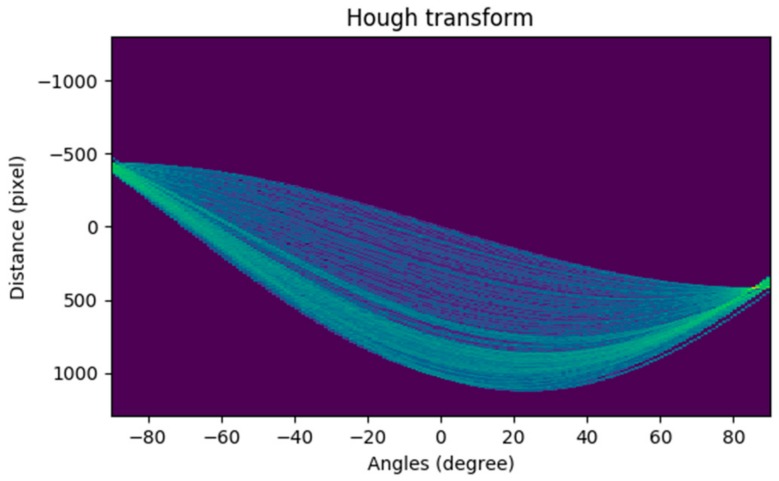
Hough space of the edge detection image.

The Hough transform is used to display the edge detection result in the accumulator. As shown in Figure 8, the horizontal coordinate represents the polar angle (θ) and the vertical coordinate represents the polar diameter (ρ), and each curve represents a point in the edge image. The brighter the point, the more the number of curves (represented by τ ) that pass through this point, indicating that the more points are collinear in the edge image. In this paper, we optimize the SSL extraction algorithm by combining the prediction results calculated by Hough transform according to the SSL length, with the measurement results provided by the inertial sensor according to the SSL angle. The specific algorithm is as follows:

1. Prediction of SSL using Hough transform. In order to avoid the sample dispersion problem caused by the excessive voting range of the accumulator, we use the Kernel-based Hough transform [34] algorithm to smooth the accumulator. First, we calculate five clusters of pixel points with collinear features, and then find the best fitting line and model the uncertainty for each cluster, and last, vote for the main lines using elliptical-Gaussian kernels computed from the lines associated uncertainties. We obtain the following parameters using Figure 7b processed by Hough transform, as shown in Table 1. In order to present five SSLs visually, we use five colors to mark them in the original image, as show in Figure 7c.
2. Measurement results with the inertial sensor. In an ideal state, the roll angle γ obtained by the inertial sensor is the tilt angle of the SSL, and there is a mutual residual between γ and θ. Therefore, we can take θ into the Hough space to find the SSL. However, there is a certain measurement error in the inertial sensor data, so it is necessary to comprehensively consider the prediction result of Hough transform and the measurement result of inertial sensor.
3. Defining cost function. Firstly, we use τ and φ to represent the prediction of length and angle, (l/cosγ) and γ represent the measurement of length and angle, ω and (1 − ω) represent the influence factors of length and angle, respectively. The cost function can be obtained from Equation (14). Then, we need to eliminate the effect of the angle’s direction on the cost function. If γ≥0, we need to remove the SSL with a negative angle in the predicted value; the processing method is the same if γ<0 . Finally, in order to eliminate the dimensional influence between the evaluation metrics, the min–max normalization processing method is used to map the result values between [0, 1], and the conversion function is shown in Equation (15).
  (14) $$J_{min}=\omega (\tau -{\left(l/\cos\gamma )\right)}^{2}+\left(1-\omega \right){\left(\varphi -\gamma \right)}^{2}$$
  (15) $$x^{*}=\frac{x-min}{max-min}$$

Assuming *l* = 1288 pixels, γ = −5.0°, and ω = 0.4, the relevant parameters of the cost function are shown in Table 2. We know SSL-1 is the optimal SSL and display it in the original image as shown in Figure 7d.

## 5. Visual Saliency Detection in the ROSD of the SSL

After obtaining the optimal SSL, we add 30 pixels to the rectangle where the optimal SSL is located, cut it, and define it as the ROSD. In the ROSD, the influence of clouds and sea clutter is small. The long-distance ship is mainly near the SSL, and the sea–sky background is relatively uniform and connected with the boundary part of the area. According to the characteristics of the ROSD, we use the fast minimum barrier distance (FMBD) [35] to measure the connectivity of the pixel and the region boundary. The algorithm operates directly on the original pixel, and does not have to acquire the superpixel of the image through the region abstraction [36,37,38,39], which improves the detection performance of the saliency map.

The FMBD algorithm mainly consists of three steps, namely, obtaining the minimum barrier distance (MBD) distance map, backgroundness, and post-processing. We used the same approach as FMBD in the first two steps, but we made appropriate improvements in the post-processing step. The specific algorithm is as follows:

Firstly, we convert the color space of the ROSD from RGB to Lab to better simulate the human visual perception. In each channel, we select a pixel-wide row and column as the seed set *S* in the upper, lower, left, and right boundaries of the ROSD region. Then, the FMBD algorithm is used to calculate the path cost function of each pixel in the ROSD region to the set *S*, as shown in Equation (16), where i represents any pixel other than the boundary in the image, and $\pi \left(i\right)$ represents the path of the pixel to the set *S*. In this paper, we consider four paths adjacent to each pixel point; $I\left(\cdot \right)$ represents the pixel value of a point, and the cost function $\beta_{I}\left(\pi \right)$ represents the distance between the highest pixel value and the lowest pixel value on a path.

(16) $$\beta_{I}\left(\pi \right)=\underset{i=\left[0,k\right]}{\max}I\left(\pi \left(i\right)\right)-\underset{i=\left[0,k\right]}{\min}I\left(\pi \left(i\right)\right)$$

We scan the ROSD area three times, which are raster scan, inverse raster scan, and raster scan. In each scan, half of the four neighborhoods of each pixel are used; that is, the upper neighborhood and the left neighborhood pixel. The path minimization operation is shown in Equation (17), where $P\left(y\right)$ represents the path currently assigned to pixel y, $\langle y,\;x\rangle$ represents the edge from pixel y to pixel x, $P\left(y\right)\cdot \langle y,\;x\rangle$ represents the path of x, and the direction is from y to x. Assuming $P_{y}\left(x\right)=P\left(y\right)\cdot \langle y,\;x\rangle$, you can get Equation (18), where $U\left(y\right)$ and $L\left(y\right)$ are the maximum and minimum values on the path, respectively.

(17) $$D\left(x\right)=\min\left\{\begin{array}{c} D\left(x\right)\\ \beta_{I}\left(P\left(y\right)\cdot \langle y,\;x\rangle \right) \end{array}\right.$$

(18) $$\beta_{I}\left(P_{y}\left(x\right)\right)=\max\left\{U\left(y\right),\;I\left(x\right)\right\}-\min L\left(y\right),I\left(x\right)$$

In summary, when a pixel appears in the region of the salient target, its pixel value should be close to the maximum pixel value on each path, and the cost function here is relatively large. When a pixel appears in the background area, its pixel value should be close to the minimum pixel value on each path, and the cost function here is relatively small. Thereby, the highlighting area can be realized, the background area can be darkened, and the target saliency detection can be completed.

Secondly, after obtaining the FMBD distance maps accumulated in the three-color spaces, we apply the backgroundness cue of the ROSD region to enhance the brightness of the saliency map. In the ROSD, the boundary of the image is the sea–sky background. According to this feature, first, we select 10% of the area in the upper, lower, left, and right directions of the ROSD as the boundary part, and then calculate the Mahalanobis Distance of the color mean between all the pixels and the four boundary areas. Finally, the maximum value of the boundary information is subtracted from the sum of the boundary information obtained from the four regions to obtain a boundary comparison map. Therefore, we can exclude the case where a region may contain a foreground region, as shown in Equation (19), where $\bar{x}$ and $Q^{-1}$ represent the color mean and covariance of each boundary part, respectively.

(19) $$\left\{\begin{array}{c} u_{k}^{ij}=\sqrt{\left(x_{k}^{ij}-\bar{x}\right)Q^{-1}{\left(x_{k}^{ij}-\bar{x}\right)}^{T}}\\ u^{ij}=\sum_{k=1}^{4}u_{k}^{ij}-\underset{k}{\max}u_{k}^{ij} \end{array}\right.$$

Finally, in the post-processing section, the three processing techniques of the original article do not adapt to ship detection near the SSL, so we make appropriate improvements. For the first processing, we replace the previous morphological filtering with morphological reconstruction with opening operation. The specific operation is that we use the structural element *b* to erode the saliency map (the saliency map is represented by F) n times to obtain the erosion map $F^{\prime }$, then use *b* to dilate $F^{\prime }$. Next, we take the minimum value of the dilation map and the original map F, and iterate the process until $F^{\prime }$ no longer changes. The results of our processing can be obtained by Equation (20), where ⊕ and ⊖ represent the dilation and erosion operations in morphology, respectively. For the second processing, the original processing utilizes the image enhancement technique in the middle of the image, but it is easy to ignore the small targets around, so this paper directly removes this technology. The third processing is consistent with the original article; the sigmoid function is used to increase the contrast between the target and the background region, as shown in Equation (21), where parameter *a* is used to control the contrast level of the target and the background.

(20) $$\left\{\begin{array}{c} F^{\prime }=\left(F⊖nb\right)\\ O_{R}^{\left(n\right)}\left(F\right)=min\left(F^{\prime }⊕b\right),F \end{array}\right.$$

(21) $$f\left(x\right)=\frac{1}{1+e^{-a\left(x-0.5\right)}}$$

**Figure 9:**
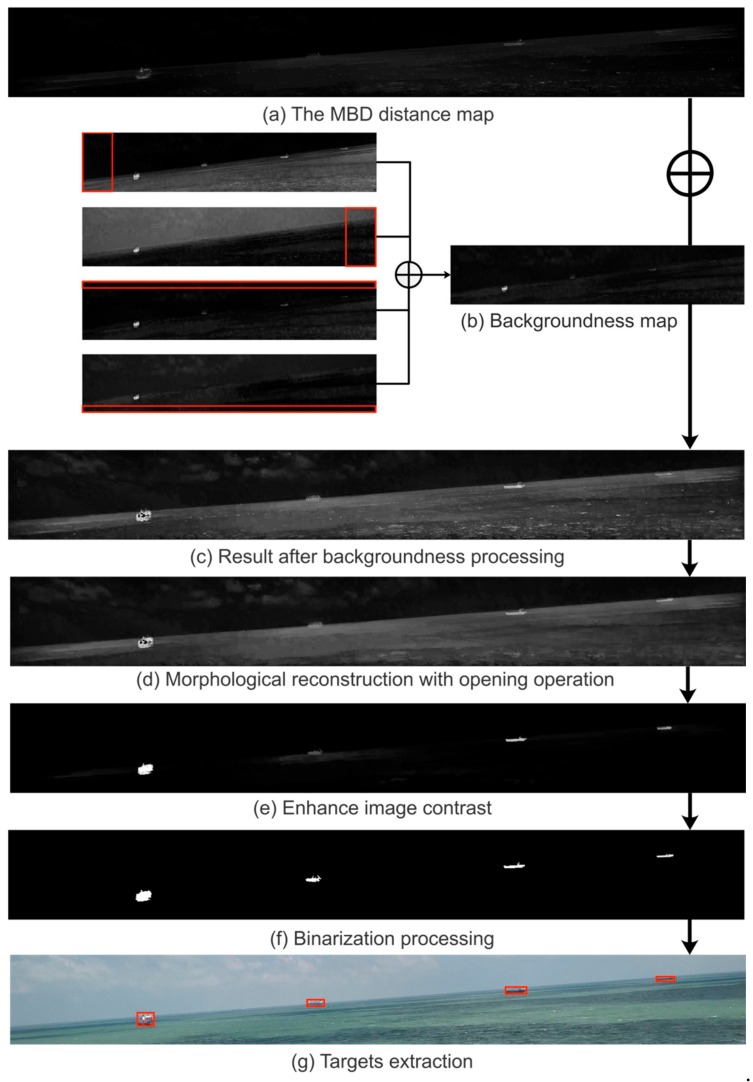
A processing case for targets detection. (**a**) is obtained by fusing the average values of the MBD maps of the three channels L, a, and b. In (**b**), each red box represents 10% of the image area in the four directions of up, down, left, and right, and ⊕ represents the average of the four images after adding. (**d**,**e**) represent post-processing, where n=8 and a=10. (**f**,**g**) represent foreground segmentation, where the binarization threshold is 5 times the average intensity of (**e**), and the area threshold is 100 pixels.

The saliency feature map obtained by the proposed algorithm has the following characteristics: The target part is highlighted, the background part is darkened, and the contrast is obvious. We select the appropriate threshold to test the saliency map, and use the area threshold to extract the final l target ship, eliminating trivial small area interference. The processing of target detection is shown in Figure 9.

## 6. Experimental Results and Discussion 

This paper conducts a real ship experiment on the “YUKUN” of Dalian Maritime University’s special teaching practice ship, and uses an inertial sensor and a visible light camera for data acquisition. The inertial sensor adopts the MTi-G-700 MEMS inertial measurement system produced by Xsens Company of the Netherlands. The measurement range of roll angle and pitch angle is [–180°, 180°], and the accuracy of measurement is less than 0.1°. The camera uses the Blackfly U3-13S2C/M-CS camera from PointGrey, Canada. The chip size is 4.8 × 3.6 mm, and the number of pixels on the target surface is 1288 × 964. The camera focal length during the experiment was 27.82 mm. All the experiments in this paper were tested on an Intel i5 processor, 8G memory MacBook Pro, and programmed in Python.

### 6.1. Dataset and Evaluation Indicators

#### 6.1.1. Dataset

For maritime target detection, there is currently no authoritative dataset to verify the validity of the algorithm. A few datasets that have been opened do not include camera-related attitude data. Therefore, the images in our dataset were obtained by the Blackfly U3-13S2C/M-CS camera installed on the “YUKUN” ship. The image size is 1288 × 964 pixels. We used the inertial sensor and visible camera synchronization processing algorithm to obtain the camera motion attitude data. The detailed information of the experiment images is shown in Table 3.

#### 6.1.2. Evaluation Metrics

As can be seen from Section 4.1, each SSL can be represented by a rectangle, so we can describe the SSL by Equation (22). The true value can be obtained by manually marking SSLs in the images. In the experiment, to verify the camera’s motion attitude model, we can calculate the difference between the estimate values and the actual values of *LC* and *H* to obtain the model accuracy. In evaluating the detection performance of the SSL, if the difference of *LC* is less than 5 pixels and the difference of *H* is less than 10 pixels, we believe that the SSL is correctly detected.

In evaluating the performance of detection, we used the confusion matrix of classification result to represent the detection results, namely, the true positive (*TP*), false positive (*FP*), true negative (*TN*), and false negative (*FN*). Precision and recall were obtained by Equation (23). Intersection over Union (IoU) was also used as the evaluation metrics; that is, the intersection of the detection result and the true value was compared to their union. When the IoU was greater than or equal to 0.5, the test result was marked as *TP*. When IoU was less than 0.5, the test result was marked as *FP*.

(22) L=[LC, H]

(23) $$P=\frac{TP}{TP+FP},\;R=\frac{TP}{TP+FN}$$

### 6.2. Experimental Results and Discussion on SSL Detection

The SSL extraction algorithm in this paper mainly includes three models, namely, camera motion attitude model, improved edge detection model, and improved Hough transform model. In order to verify the performance of each model separately, the following three experiments were designed. Some parameter settings in the experiment are shown in Table 4.

1 Experiment 1—Verification of the camera motion attitude model

The camera motion attitude model uses the pitch angle β and roll angle γ provided by the inertial sensor to estimate the CR of the SSL in the image, which can effectively narrow the detection range and is of great significance for subsequent algorithms. In this experiment, we used the difference between *LC* and *H* of the SSL candidate region and the real region to describe the estimation accuracy. 

Eight experiment results of the model accuracy are shown in Table 5. The total number of detected images is 2000. The analysis results show that the *LC* estimation accuracy of the camera motion attitude model is 6–13 pixels, and the *H* estimation accuracy is 7–19 pixels. It can be seen from the experimental results that it is reasonable to estimate the rectangular area of the SSL by using the camera motion attitude model, and then increase the height of 30 pixels above and below the estimate rectangular as the CR of the SSL, which can effectively ensure that the real SSL is in the CR.

2 Experiment 2—Verification of the improved edge detection model

**Figure 10:**
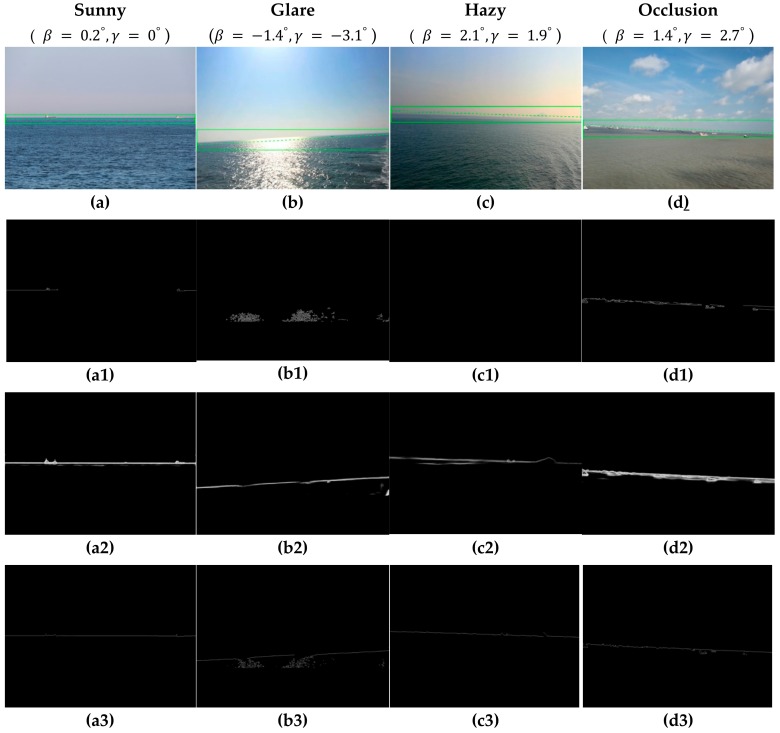
Edge detection results by the four method. (**a**–**d**) Original images with CR. (**a1**–**d1**) Edge detection results by Canny (30,150). (**a2**–**d2**) Edge detection results by HED. (**a3**–**d3**) Edge detection results by the proposed method.

This experiment was carried out in sunny, glare, hazy, and occlusion conditions from the train set, and compared the performance with the Canny operator and the deep learning-based holistically-nested edge detection (HED) algorithm [40]. In order to better illustrate the detection performance of various algorithms, one image was selected for description under four conditions, as shown in Figure 10. 

First of all, we used the data provided by the inertial sensor to obtain the CR of the SSL through the camera motion attitude model, as shown in Figure 10a–d, then used three algorithms to process the image separately. We can see that the Canny operator had the worst detection performance of the SSL, since the threshold was not adaptive. In sunny conditions, only part of SSL could be detected, as shown in Figure 10(a1). When the glare conditions or the sea–sky background was hazy, the Canny operator failed to detect the edge of SSL, as shown in Figure 10(b1,c1). When there were obstacles such as ships or islands blocking the SSL, the Canny operator detected the edge of the obstacle and this affected the performance, as shown in Figure 10(d1). The HED algorithm achieved better performance under any condition, but it was easy to cause over-detection. In addition to identifying the SSL, sea clutter was detected in Figure 10(c2), and the edge of the obstruction was added to the SSL in Figure 10(d2). The proposed algorithm achieved the best performance, since the binary division was performed in the adjacent small blocks, over-detection was effectively prevented while ensuring the threshold adaptive. Although part of the spot was detected in Figure 10(a3), it did not affect the extraction of the SSL.

3 Experiment 3—Verification of the improved Hough transform model

**Figure 11:**
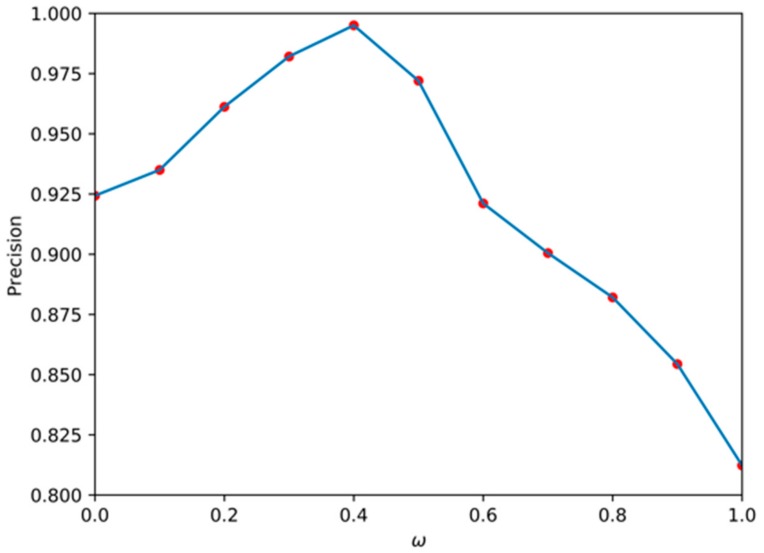
Relationship between the average precision (AP) of SSL detection and ω.

This experiment was mainly to verify the effect of length and angle on the cost function at the stage of SSL extraction. In the train set, we set the value of ω to [0, 0.1, 0.2, 0.3, 0.4, 0.5, 0.6, 0.7, 0.8, 0.9, 1.0], found the minimum cost function for each value, and drew the corresponding SSL. According to the evaluation metrics of the SSL, the average precision (AP) of the SSL under each ω is shown in Figure 11. It can be seen that when ω = 0.4, the extracted SSL had the highest AP, reaching 99.5%, but when only the length factor of the SSL was considered, the AP was the lowest with just 81.23%. Therefore, it can be concluded that the influence of the angle is greater than the length in the cost function extracted by the SSL.

The above three experiments were fully verified for each model of the SSL detection. According to the proposed algorithm, we processed 600 images in the test set, and compared with Fefilatyev’s method [7] and Zhang’s method [10]. The precision and recall rates are as shown in Table 6. We can see that all of the three methods achieved good performance in SSL detection, but the proposed method was still better than the other two methods, whose average precision (AP) and average recall (AR) in the test set reached 99.67% and 100%, respectively.

### 6.3. Experimental Results of Ship Detection in the Train Set

In this experiment, there were a total of 1050 images in the train set. In order to verify the performance of the proposed method, the other three saliency detection algorithms, SR [41], RBD, and traditional FMBD, were used as the comparison experiments. The parameters of the proposed method are in Table 7.

When evaluating the performance of ship detection, the binarization threshold *T* and the area threshold *S* determine the satisfaction from two aspects of the pixel intensity and the number of pixels connected. If the *T* and *S* are too large, the target will be submerged in the background. If the *T* and *S* are too small, false target interference will occur. When determining the range of *T*, the target pixel intensity significantly exceeds the average intensity $\bar{T}$, so the value of *T* represents by times of the average intensity ($\bar{T}$) of the salient map, and a combination of *T* and *S* is shown in Table 8.

**Figure 12:**
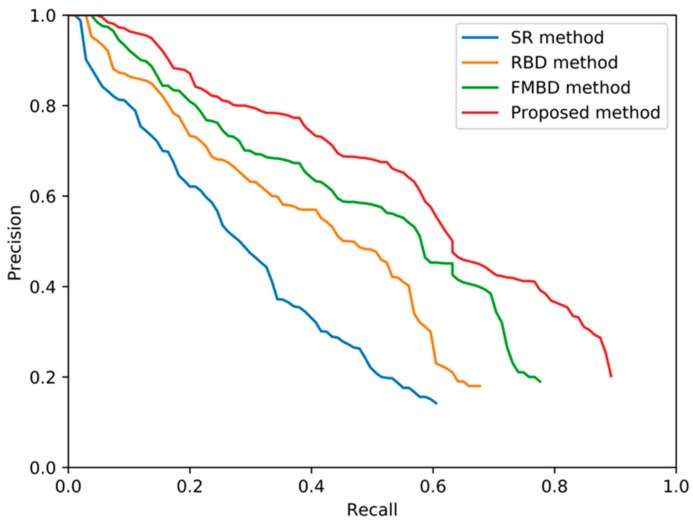
Precision-recall curves of the four object detection methods.

According to the above threshold combination, we can draw the precision-recall graphs of the four detection methods, as shown in Figure 12. It can be seen that the proposed method is superior to the other three saliency detection methods.

**Figure 13:**
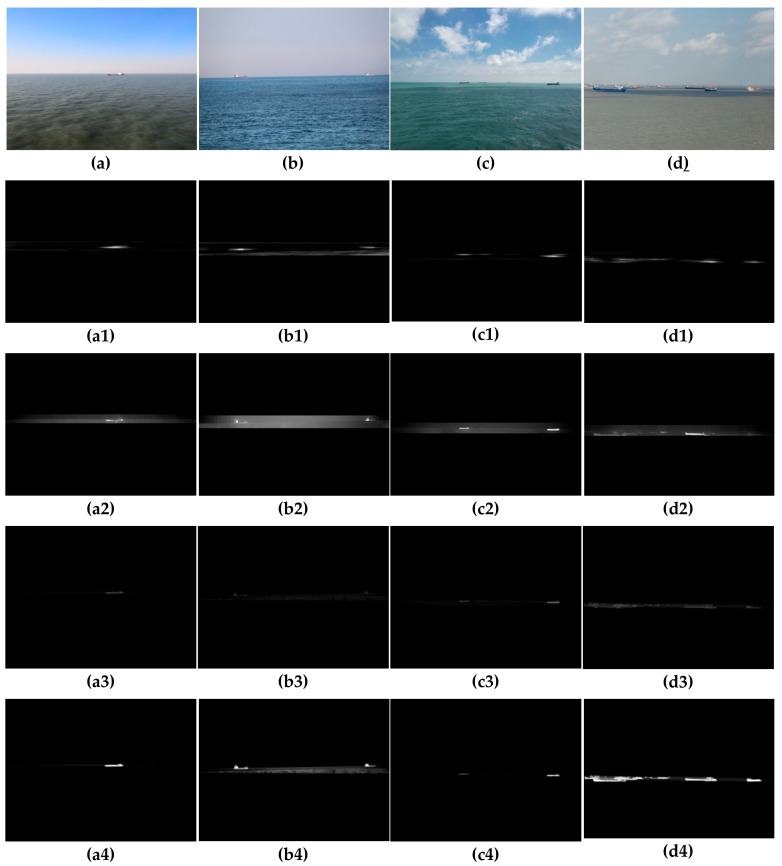
Saliency detection results by the four methods. (**a**–**d**) Original images. (**a1**–**d1**) Saliency detection results by SR. (**a2**–**d2**) Saliency detection results by RBD. (**a3**–**d3**) Saliency detection results by FMBD. (**a4**–**d4**) Saliency detection results by the proposed method.

Figure 13 shows the detection performance of several methods on different datasets of the train set. It can be seen that although the residual spectrum (Figure 13(a1–d1)) obtained by the SR method can detect the ship, it does not accurately indicate the position and shape of the target ship, and is liable to cause false detection. The RBD method achieves better detection results when the number of targets in the image is small, as shown in Figure 13(a2,b2), but it is easy to cause missed detection when there are many targets, such as Figure 13(c2,d2). The traditional FMBD method (Figure 13(a3–d3)) can detect the salient targets well, but the contrast between the target and the background is not obvious enough, which is not conducive to subsequent target extraction. The proposed method (Figure 13(a4–d4)) in this paper can clearly distinguish the target and background, accurately detect the shape and position of the target, and the detection performance is the best.

**Figure 14:**
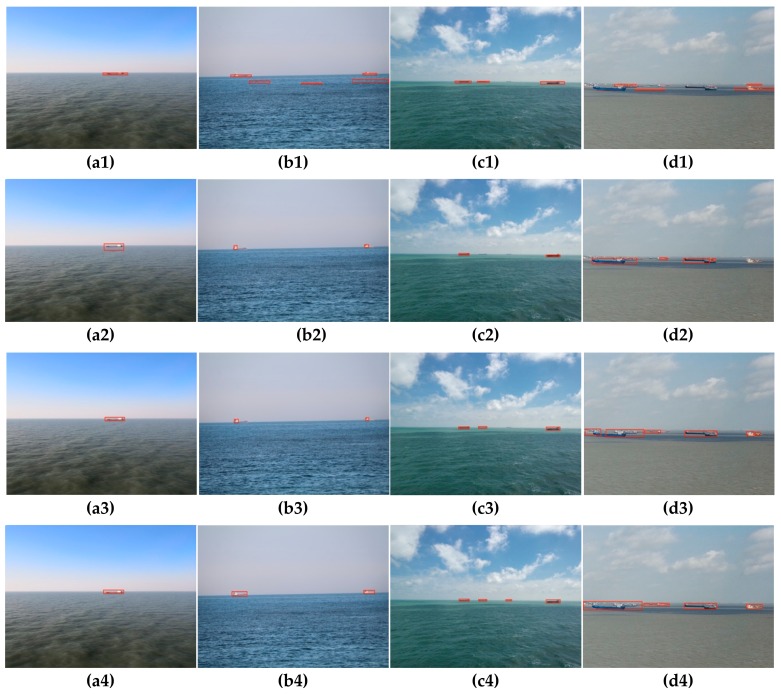
Object segmentation results by the four methods. (**a1**–**d1**) Object segmentation results by the SR method. (**a2**–**d2**) Object segmentation results by the RBD method. (**a3**–**d3**) Object detection results by the FMBD method. (**a4**–**d4**) Object segmentation results by the proposed method.

Figure 14 shows segmentation results of the target ships from the salient feature map where the *T* is 5 times of $\bar{T}$ and *S* is set to 100 pixels. It can be seen from Figure 14(a1–d1) that the SR method has the worst segmentation result, the detection result has many missed detections and false detections, and the target ship positioning accuracy is also poor. The RBD method is more powerful than the SR method, but with a certain degree of target missed detection, such as Figure 14(c2,d2). The traditional FMBD method has a good segmentation result, but it still has some shortcomings in target positioning accuracy and missed detection, such as Figure 14(a3–c3). The proposed method accurately detects the target in Figure 14(a4–c4), but the method fails to perform accurate target segmentation when the target ship appears to be covered, as shown in Figure 14(d4). This is also a direction we will focus on in the future.

### 6.4. Experimental Results of Ship Detection in the Test Set

In this experiment, we verified the proposed target detection method in the test set and compared it with Fefilatyev’s and Zhang’s methods. The precision and recall rates are shown in Table 9. Since Fefilatyev’s method only detects the ship above the SSL, both AP and AR are relatively low. Zhang’s method is relatively good, as the AP and AR reached 59.21% and 73.25%. However, the proposed method achieved the best scores, with an AP and AR of 68.50% and 88.32%, respectively.

## 7. Conclusions

This paper proposes a novel maritime target detection algorithm based on the motion attitude of visible light camera. The camera was fixed on the “YUKUN” ship, and the camera’s motion attitude data was acquired synchronously by the inertial sensor, so that the CR of the SSL on the image could be estimated. Then, the improved local Otsu algorithm was applied to the edge detection in the CR, and the Hough transform was improved to extract the optimal SSL. Finally, the improved FMBD algorithm was used to detect the target ships in the vicinity of the SSL. The experimental results show that the proposed algorithm has obvious advantages compared with the other maritime target detection algorithms. In the test set, the detection precision of the SSL reached 99.67%, effectively overcoming the complex maritime environment. The ship detection precision and recall rates were 68.50% and 88.32%, respectively, which improved the detection precision while avoiding the ship’s missed detection.

The main contribution of this paper is the construction of a camera motion attitude model by analyzing the six-degrees-of-freedom motion of the camera at sea, combined with the maritime target detection algorithm, which narrowed the detection range and improved the detection accuracy. The edge detection algorithm was improved. The local Otsu algorithm was used for edge processing in the CR, which effectively overcame the complex maritime environment. The Hough transform algorithm was improved. The length and angle of the SSL were simultaneously considered as evaluation metrics of the cost function, which effectively improved the accuracy of SSL extraction. The ROSD was detected by the improved the FMBD algorithm. In the post-processing part of the algorithm, the morphological reconstruction with opening operation, was used to replace the previous processing method to smooth the sea–sky background, which effectively improved the target ship’s saliency detection effect.

## Figures and Tables

**Table 1:** Hough spatial parameters of candidate SSLs ranked top five.

Rank	(θ, ρ)	τ
SSL-1	(85.90°, 528.66)	606
SSL-2	(86.92°, 512.66)	271
SSL-3	(87.95°, 557.67)	183
SSL-4	(83.85 °, 572.68)	165
SSL-5	(82.82 °, 592.68)	162

**Table 2:** The relevant parameters of the cost function.

Rank	τ	$\left(l/\cos\gamma -\tau \right)*$	φ (°)	$\left(\gamma -\varphi \right)*$	$J_{min}$
SSL-1	606	0	4.10	0	0
SSL-2	271	0.75	3.08	0.50	0.38
SSL-3	183	0.95	2.05	1	0.96
SSL-4	165	0.99	6.15	0.12	0.40
SSL-5	162	1	7.18	0.62	0.63

**Table 3:** Detailed information of the experiment images.

Type of Images	Conditions	Train Set	Test Set	Total
Images with SSL	Sunny	350	150	500
	Glare	350	150	500
	Hazy	350	150	500
	Occlusion	350	150	500
Images with ships	One ship	420	180	600
	Two ships	210	90	300
	Multiple ships	280	120	400
	Multiple ships + Occlusion	140	60	200

**Table 4:** Some parameter settings in the test.

Parameter	Description	Value
*h*(m)	The height of the camera position	20
*l*(pixel)	The width of the image	1288
*w*(pixel)	The height of the image	964
*N*	Number of adjacent image blocks in local Otsu algorithm	16

**Table 5:** Location estimated accuracy of camera motion attitude model.

Dataset	Number of Images	Mean Difference of *LC* Coordinate (Pixels)	Mean Difference of *H* (Pixels)
Sunny (Train)	350	7.09	8.82
Glare (Train)	350	8.53	13.46
Hazy (Train)	350	6.09	7.14
Occlusion (Train)	350	11.45	18.26
Sunny (Test)	150	6.81	10.55
Glare (Test)	150	9.41	17.32
Hazy (Test)	150	12.75	18.89
Occlusion (Test)	150	10.46	14.21

**Table 6:** Precision and recall scores for the three methods.

Dataset (Test)	Number of Images	Fefilatyev’s Method (%)		Zhang’s Method (%)		Proposed Method (%)	
		P	R	P	R	P	R
Sunny	150	100	100	100	100	100	100
Glare	150	91.10	97.08	97.67	85.71	100	100
Hazy	150	94.37	94.37	99.28	91.95	99.33	100
Occlusion	150	96.62	98.62	98.58	93.92	99.33	100
Total	600	95.56	97.56	98.75	92.91	99.67	100

**Table 7:** Experiment parameters of saliency detection.

Parameter	Description	Value
*n*	Number of erode operations in post-processing	8
*a*	The parameter of function sigmoid in post-processing	10

**Table 8:** Combination of *T* and *S.*

Threshold	Value									
*T* ($\bar{T}$)	1	2	3	4	5	6	7	8	9	10
*S* $\left({\mathrm{pixel}}^{2}\right)$	20	40	60	80	100	120	140	160	180	200

**Table 9:** Precision and recall scores of the three ship detection methods.

Dataset (Test)	Number of Images	Number of Ships	Fefilatyev’s Method (%)		Zhang’s Method (%)		Proposed Method (%)	
			AP	AR	AP	AR	AP	AR
Total	450	1023	19.15	35.26	59.21	73.25	68.50	88.32
